# Targeted Central Nervous System Irradiation of *Caenorhabditis elegans* Induces a Limited Effect on Motility

**DOI:** 10.3390/biology9090289

**Published:** 2020-09-14

**Authors:** Michiyo Suzuki, Zu Soh, Hiroki Yamashita, Toshio Tsuji, Tomoo Funayama

**Affiliations:** 1 Department of Radiation-Applied Biology Research, Takasaki Advanced Radiation Research Institute, National Institutes for Quantum and Radiological Science and Technology (QST-Takasaki), 1233 Watanuki, Takasaki, Gunma 370-1292, Japan; funayama.tomo@qst.go.jp; 2 Electrical, Systems, and Control Engineering Program, Graduate School of Advanced Science and Engineering, Hiroshima University, 1-4-1 Kagamiyama, Higashi-Hiroshima, Hiroshima 739-8527, Japan; sozu@hiroshima-u.ac.jp (Z.S.); tsuji@bsys.hiroshima-u.ac.jp (T.T.); 3 Department of System Cybernetics, Graduate School of Engineering, Hiroshima University, 1-4-1 Kagamiyama, Higashi-Hiroshima, Hiroshima 739-8527, Japan; yamasita@bsys.hiroshima-u.ac.jp

**Keywords:** central nervous system, targeted irradiation, microbeam, carbon ions, microfluidic chip, on-chip imaging analysis, motility, *Caenorhabditis elegans*

## Abstract

To clarify the tissue responsible for a biological function, that function can be experimentally perturbed by an external stimulus, such as radiation. Radiation can be precisely and finely administered and any subsequent change in function examined. To investigate the involvement of the central nervous system (CNS) in *Caenorhabditis elegans’* locomotion, we irradiated a limited 20-µm-diameter area of the CNS with a single dose and evaluated the resulting effects on motility. However, whether irradiated area (beam size)-dependent or dose-dependent effects on motility occur via targeted irradiation remain unknown. In the present study, we examined the irradiated area- and dose-dependent effects of CNS-targeted irradiation on the motility of *C. elegans* using a collimating microbeam system and confirmed the involvement of the CNS and body-wall muscle cells around the CNS in motility. After CNS-targeted microbeam irradiation, *C. elegans’* motility was assayed. The results demonstrated a dose-dependent effect of CNS-targeted irradiation on motility reflecting direct effects on the irradiated CNS. In addition, when irradiated with 1000-Gy irradiation, irradiated area (beam size)-dependent effects were observed. This method has two technical advantages: Performing a series of on-chip imaging analyses before and after irradiation and targeted irradiation using a distinct ion-beam size.

## 1. Introduction

The nematode *Caenorhabditis elegans* (*C. elegans*), a powerful model for studying many biological mechanisms, such as development, aging, and nervous system function, has only 959 somatic cells and approximately 2000 germ cells in the hermaphrodite but has various behaviors such as stimulation responses, learning and memory, and motor control [1,2,3]. We focused on the principle of muscle movements in *C. elegans*. An important issue is to identify the rhythm generator, a “control tower” of harmonious and rhythmic locomotion (forward and backward motions), by clarifying the functions and intercellular interactions of individual cells. This can be achieved by perturbing biological functions using external stimuli and recording responses that differ from normal. We selected radiation, which can be controlled precisely and finely, as an external stimulus, and investigated which biological functions in *C. elegans* changed under its effect.

We previously found that motility (locomotion) in *C. elegans* was significantly reduced by whole-body irradiation with ^60^Co gamma rays or heavy ions, in a dose-dependent manner [4,5]. To investigate which cell and tissue responses of the multicellular system reflected the response after whole-body irradiation, we targeted irradiation to a limited area with a heavy-ion microbeam as a radiosurgery technique [6,7,8,9]. When using the microbeam, we found that targeted irradiation of any individual region, including the central nervous system (CNS), with at least 500 Gy did not affect motility. This suggested that radiation inhibits locomotion by a whole-body mechanism, potentially involving motor neurons and/or body-wall muscle cells, rather than affecting motor control via the CNS or a stimulation response [10]. Locomotion, such as forward motion, backward motion, and turns, is accomplished in *C. elegans* by body-wall muscle cells located from the head to the tail, which are controlled by motor neurons in the ventral nerve cord. In contrast, most sensory integration occurs in a nerve ring (as in the CNS) wrapped around the pharynx in the head. Pharyngeal muscle cells pump food into the gut and are not considered to be involved in locomotion [11].

Despite the finding that no effect on motility was observed immediately after CNS-targeted irradiation with 500 Gy [10], we questioned the involvement of the CNS in the motility response to radiation. In previous experiments, we targeted a limited area with a diameter of 20 µm and irradiated it with a single dose of 500 Gy (a dose that reduced motility after whole-body irradiation [5,10]); therefore, whether irradiated area (beam size)-dependent and dose-dependent effects on motility by targeted irradiation occur remain unknown. Because the CNS is a vital tissue, the effects of targeted irradiation should be examined in more detail. Evidence of dysfunction in neurons, such as motor neurons located in the anterior part of the nerve cord [12,13,14], indicate CNS-targeted irradiation may induce effects on the neuronal control of motility.

**Figure 1:**
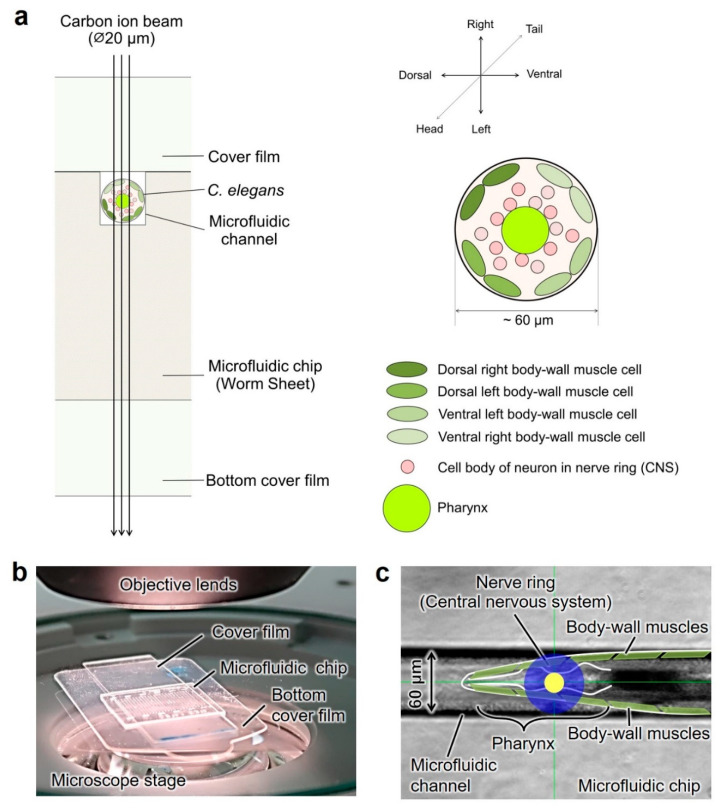
Schematic of the CNS-targeted irradiation of individual live *C. elegans* using a microfluidic chip. (**a**) Cross-sectional view of CNS-targeted irradiation of a live *C. elegans* enclosed in a microfluidic channel of a microfluidic chip. Anatomical structure of an individual *C. elegans* in the right panel was compiled with reference to a previous report [3]. (**b**) Schematic of a 300-µm, ultra-thin, ion-penetrable, wettable, microfluidic chip (Worm Sheet IR) enclosing live *C. elegans* individuals in the microfluidic channels. (**c**) Overhead view of the anterior body of an individual live *C. elegans* enclosed in a straight microfluidic channel of a microfluidic chip. The whole nerve ring in a ∅60-µm micro-aperture region (filled in blue) or the center of the nerve ring (CNS) in a ∅20-µm micro-aperture region (filled in yellow) were targeted and irradiated with an exact number of carbon ions.

In the present study, we aimed to examine the irradiated area- and dose-dependent effects of CNS-targeted irradiation on the motility of *C. elegans* using a collimating microbeam system at QST-Takasaki (Gunma, Japan) [15]. We previously developed an ion-penetrable, ultra-thin polydimethylsiloxane (PDMS) microfluidic chip (Figure 1), named the Worm Sheet, for on-chip immobilization for targeted microbeam irradiation of individual live *C. elegans* [16]. Here, we propose a novel analysis that combines targeted irradiation with an on-chip behavioral imaging approach using a Worm Sheet that allows the rapid, direct, multiplex measurement of individual animals before and after irradiation. There are two technical advantages in the present study. One is the series of on-chip imaging analyses before and after irradiation and the other is targeted irradiation using a distinct ion-beam size. These two points are outlined below.

On-chip immobilization techniques play an important role in experiments involving targeted irradiation. The thickness of conventional PDMS microfluidic chips developed for *C. elegans* is generally over 2 mm and does not allow heavy-ion particles to pass through; therefore, ion particles hitting animals are not detected by an ion detector located under the sample stage of a collimating microbeam system [15]. To count ions hitting animals in good active condition during irradiation, we recently developed an ion-penetrable, ultra-thin, PDMS microfluidic chip (Figure 1), named the Worm Sheet, which permits careful control of the number of ions (dose) applied [16]. The thickness of a sample, which includes the microfluidic chip enclosing the animal and the upper and bottom cover films, was only 550 µm, thus enabling carbon ions (whose range in water is only 930 µm) to pass through (Figure 1a). Moreover, this PDMS microfluidic chip has wettability that allows animals enclosed in the microfluidic channels to be maintained in good active condition. By using the Worm Sheets, we conducted the targeted irradiation of the CNS in *C. elegans* with an exact number of heavy-ion particles, specifically carbon ions. Furthermore, this system can replace various micro-aperture cylinders with holes of a specific size (e.g., 20-, 120-, or 250-µm diameter) to form ion beams of a specific size. In addition to a micro-aperture (beam exit) with a 20-µm diameter used in previous studies [10,16], we used a new micro-aperture of 60-µm diameter (equal to the body width of *C. elegans*), which can apply irradiation consisting of carbon ions, whose range is less than 1 mm in water. Using the two micro-apertures, we investigated the area-dependent effects of targeted irradiation. Furthermore, to address dose-dependent effects, we developed a targeted irradiation method for *C. elegans* with an exact number of heavy-ion particles.

The increase in efficiency is important because the opportunity to perform microbeam irradiation experiments using cyclotrons is extremely limited and cannot be conducted frequently. The proposed method of microbeam irradiation with an exact number of particles enables us to irradiate a specific region of the body to analyze the immediate effects of irradiation in multiple active animals. In particular, the Worm Sheet allows experiments to be performed with efficient irradiation and observation of multiple series of animals. In this study, we show the first results of irradiation experiments on *C. elegans* using Worm Sheets.

## 2. Materials and Methods 

### 2.1. Strains and Culture

*Caenorhabditis elegans* wild-type and HBR4:*goeIs3[pmyo-3::GCamP3.35::unc-54-3’utr,unc-119(+)]V* [17] (HBR4) strains and *Escherichia coli* strain OP50 were obtained from the Caenorhabditis Genetics Center (The University of Minnesota, Minneapolis, MN, USA). *Caenorhabditis elegans* hermaphrodites were grown at 20 °C on a 6-cm plate (IWAKI nontreated dish; AGC Techno Glass, Shizuoka, Japan) containing 10 mL of nematode growth medium (NGM) spread with overnight-incubated *E. coli* [1]. Well-fed adult animals, approximately three days after hatching, were used in all experiments.

### 2.2. Evaluation of Carbon-Ion Collimation

We investigated the effects of targeted microbeam irradiation using carbon ion (^12^C^6+^) particles accelerated by an azimuthally varying field cyclotron installed at the Takasaki Ion Accelerators for Advanced Radiation Application (TIARA) facility of QST-Takasaki. Irradiation generally followed previously described methods [16,18]. We delivered targeted microbeam irradiation using a collimating microbeam system [18]. Before biological experiments using *C. elegans*, we evaluated the distribution of the collimated carbon-ion beam using a micro-aperture with a 60-µm and 20-µm diameter. We irradiated an ion-track detector CR-39 film (Solid State Nuclear Track Detector HARZLAS TNF-1, Fukuvi Chemical Industry, Fukui, Japan) with 10 or 100 ion particles and observed the resulting etch pits as described previously [10,16].

### 2.3. Sample Preparation Immediately before Irradiation

**Figure 2:**
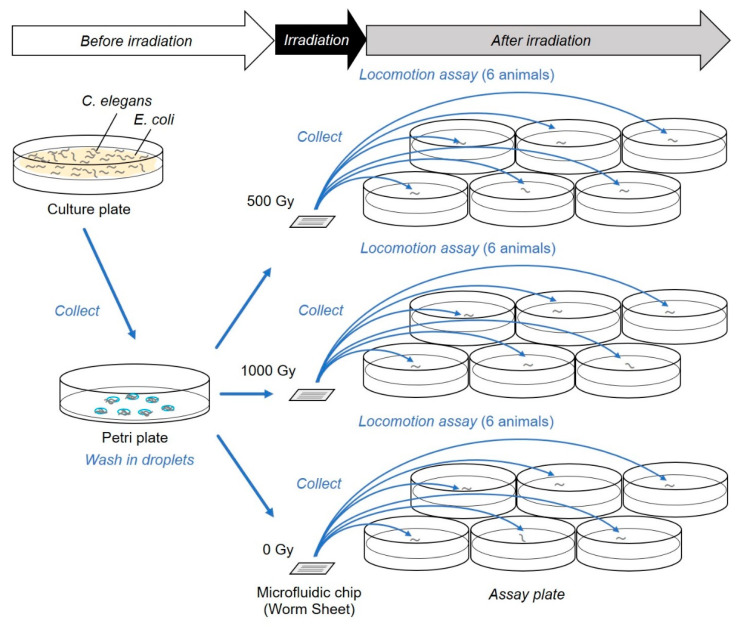
Schematic depiction of the experimental method. The procedure of the targeted-irradiation and locomotion assay using independent microfluidic chips for each dose of irradiation and assay plates for each animal. This was used in the experiments described in Section 3.1.

Sample preparation before irradiation generally followed a previously described method [18] with some improvements. Briefly, multiple *C. elegans* individuals were collected from the culture plate using a platina picker (WormStuff Worm Pick, Genesee Scientific Corporation, San Diego, CA, USA) and washed twice in drops on a nontreated plate (Figure 2). As an improvement on the previous irradiation method, we employed our developed PDMS ultra-thin, wettable, microfluidic chip (Worm Sheet IR; Biocosm Inc., Hyogo, Japan) to enclose multiple animals simultaneously. Furthermore, as we found that the use of a gelatin-based wash buffer solution (containing 5 mL of 1 M potassium phosphate (pH 6.0), 1 mL of 1 M CaCl_2_, 1 mL of 1 M MgSO_4_, and 0.5 g gelatin in 1 L of H_2_O; sterilized by autoclaving) [19] could prevent dehydration of *C. elegans* [16], we used it instead of the S basal buffer solution employed in previous CNS-targeted irradiation experiments [10].

The surface of this microfluidic chip included 25 straight microfluidic channels (70-µm depth, 60-µm width) [16,18]. Six or more washed animals for each irradiation condition were picked up using a platina picker and transferred to drops of wash buffer solution placed on the surface of each chip. To maintain humidity, a transparent cover film was placed carefully over the chip and pressed gently over the channel from one end of the chip to the other (Figure 1b). Animals were immobilized during irradiation in good active condition without the need for anesthesia. Microchip-enclosed animals were located on a bottom cover film that was placed on an aluminum frame custom made for the microbeam irradiation facility [15].

### 2.4. Targeted Microbeam or Whole-Body Broad-Beam Irradiation with High Linear Energy Transfer Carbon Ions

We investigated the effects of CNS-targeted microbeam irradiation using carbon ion particles. Irradiation generally followed a previously described method [10,16,18]. Targeted microbeam irradiation was delivered using a collimating microbeam system [15]. Briefly, the PDMS microfluidic chip enclosing multiple animals in the custom-made aluminum frame was placed on the sample stage of the collimating microbeam irradiation system and moved up to immediately under the beam exit [18]. The theoretical energy loss of carbon ion particles and linear energy transfer (LET) in *C. elegans* were calculated using the Energy Loss Modify (ELOSSM) code, part of the Induced Radioactivity Analysis Code System (IRACM) [20]. We used a track-averaged LET of 125 keV/µm in animals in microfluidic channels, and converted dose in Gy to particle number using the following relationship: Particle number = Dose (Gy)/1.6 × 10^−9^/LET (keV/µm) × Spot size (cm^2^). The nerve ring (representing the entire CNS) was targeted and irradiated with carbon ions. The whole nerve ring at a ∅60-µm micro-aperture region or the center of the nerve ring at a ∅20-µm micro-aperture region was targeted and irradiated with an exact number of carbon ions (Figure 1a,c and Table 1).

For the locomotion assay, in the case of irradiation using a ∅60-µm micro-aperture, 95,000 particles corresponding to a dose of 500 Gy or 190,000 particles corresponding to a dose of 1000 Gy were irradiated. For irradiation using a ∅20-µm micro-aperture, 10,000 particles corresponding to a dose of 500 Gy or 20,000 particles corresponding to a dose of 1000 Gy were irradiated. Six or more animals enclosed within each microfluidic chip were independently irradiated in sequence. It took approximately 10 min to irradiate six animals enclosed in a microfluidic chip with 500 Gy using a ∅60-µm micro-aperture. For comparison, we also conducted whole-body broad-beam irradiation. The track-averaged LET of the carbon-ion broad beam passing through *C. elegans* was 132 keV/µm. The microfluidic chip enclosing animals was located on a 6-cm nontreated plate, and the entire plate was irradiated with a scan beam at a dose of 500 Gy or 1000 Gy. It took approximately 50 s to irradiate each plate with 500 Gy. For all irradiation experiments, nonirradiated control animals were handled in parallel with irradiated animals, except in terms of carbon-ion irradiation. 

Moreover, for imaging muscle activities before and after CNS-targeted irradiation, four or more animals were irradiated using a ∅60-µm micro-aperture for each dose, in which 95,000 particles corresponding to a dose of 500 Gy, 190,000 particles corresponding to a dose of 1000 Gy, or 280,000 particles corresponding to a dose of approximately 1500 Gy were irradiated. It took approximately 25 min to irradiate 12 animals enclosed in a microfluidic chip with 500 Gy, 1000 Gy, or 1500 Gy using a ∅60-µm micro-aperture.

### 2.5. Locomotion Assay

The procedure is illustrated in Figure 2. Immediately after irradiation, we removed the cover film from the surface of each PDMS microfluidic chip and added a drop of wash buffer solution to multiple individuals. The animals swimming in drops were then picked up using a platina picker and placed on a freshly prepared 3.5-cm plate (IWAKI nontreated dish; AGC Techno Glass) containing of 3 mL of NGM without bacteria (food). We evaluated the motility of animals immediately after irradiation using a locomotion assay, as previously described [10], but we did not use a digital camera video recorder. Briefly, approximately five min after transfer, motility was evaluated using “body bends” [21], defined as the mean value of bends in the anterior body region at 20-s intervals in six wild-type animals, which were counted manually using a stereomicroscope (SZX7, Olympus Corporation, Tokyo, Japan) in six animals for each group and averaged. Statistical analysis was performed based on a previous method [10]. Numbers of body bends were averaged from six animals in each irradiation group. The values from five independent irradiation experiments, conducted on distinct days, were averaged and used for evaluation of the effects of irradiation. Numerical values are presented as values with standard error of the mean (SEM). To evaluate dose dependency, data were analyzed by one-way analysis of variance (ANOVA). In addition, to evaluate the main effect of the CNS-targeted irradiation, data were analyzed by two-way factorial ANOVA. This statistical analysis was applied to evaluate the existence of independent significant effects of irradiated area (beam size) and dose. All statistical analyses were conducted using Microsoft Excel software (Microsoft, Redmond, WA, USA) at 0.05 and 0.01 significance levels.

### 2.6. Series of Observations and Irradiation on a Microfluidic Chip

**Figure 3:**
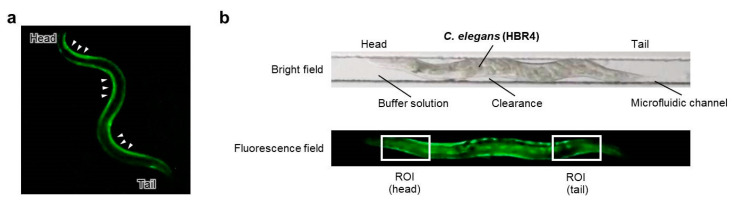
Schematic of the fluorescent imaging of body-wall muscles for *C. elegans* crawling (HBR4 strain). (**a**) Muscle contraction pattern of body-wall muscle cells during forward motion (crawling). High fluorescence (Ca^2+^) indicates the contraction of body-wall muscle cells (indicated by white triangles). (**b**) Schematic of on-chip observations of an individual *C. elegans* in a straight microfluidic channel of a Worm Sheet. The upper panel is a bright-field image of the microfluidic channel enclosing an individual active *C. elegans*, while the bottom panel is a corresponding fluorescence field image. The small degree of clearance between the body of an individual *C. elegans* and the straight microfluidic channel makes it possible for the animal to bend, such that muscle contraction and extension during crawling can be observed. To evaluate the luminance of Ca^2+^, a ROI in the head and tail area was set as a box 120 µm wide and 60 µm high.

**Figure 4:**
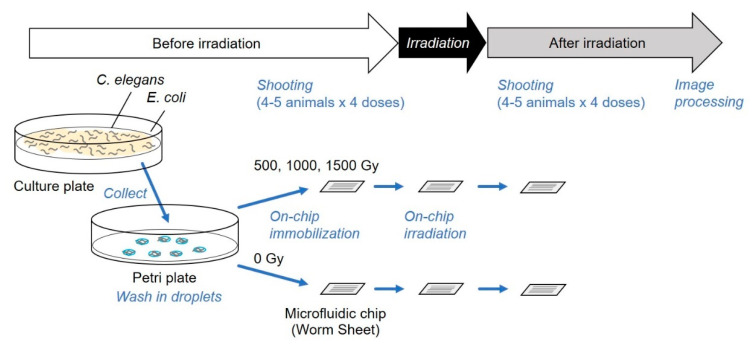
Schematic depiction of the improved experimental method. The targeted irradiation and imaging assay were completed entirely on a microfluidic chip. This was used for the experiments described in Section 3.2.

On-chip imaging observations using proposed in a previous study [18] were employed. To observe muscle contraction pattern of body-wall muscle cells during forward motion (crawling), the HBR4 strain [17] was used (Figure 3a). Young adults, which are thinner than the width of the microfluidic channels (60 μm) of the Worm Sheet, were used for imaging observations. The small degree of clearance between the body of an animal and the straight microfluidic channel of the microfluidic chip allowed animals to bend slightly, making it possible to observe muscle contraction and extension during crawling [18] (Figure 3b). The procedure is illustrated in Figure 4. For the irradiation group, 12 or more washed animals were transferred to drops of wash buffer solution placed on the surface of a microfluidic chip and enclosed. Similarly, for the non-irradiation control group, four or more washed animals were transferred to a drop on the surface of another chip and enclosed. Before irradiation, muscle contraction in the microfluidic channel was video recorded at 29.97 frames per second for approximately 1 min using a digital camera (High-Speed EXILIM, Casio Computer Co., Ltd., Tokyo, Japan) mounted on a fluorescence stereomicroscope (SZX16, Olympus Corporation, Tokyo, Japan). Subsequently, the CNS was irradiated with carbon ions and muscle contraction was video recorded at 29.97 frames per second for approximately 1 min after irradiation. Since animals can be viably maintained in a microfluidic channel of a Worm Sheet for at least three hours [16], all the irradiation experiments and observations in the present study were conducted within approximately three hours.

To evaluate Ca^2+^ fluorescence corresponding to the contraction of body-wall muscle cells before and after CNS-targeted irradiation of a ∅60-µm micro-aperture region, luminance in the head or tail region was obtained from every image by image processing. First, the ROI (head or tail) was set as a box 120 µm wide and 60 µm high in the head region (Figure 3b), which included the CNS and body-wall muscle cells surrounding the CNS, and then luminance of the ROI was obtained from 1799 fluorescent images for 1 min. Image processing for luminance analysis was conducted using MATLAB software (MATLAB ver. R2020a, The MathWorks, Inc., Natick, MA, USA). Furthermore, to evaluate changes in muscle contraction before and after irradiation, the luminance of body-wall muscle cells around the CNS or tail was calculated. Calcium ion luminance of ROI (head/tail) was calculated for individual *C. elegans* HBR4 before and after CNS-targeted irradiation of a ∅60-µm micro-aperture region. Luminance in each of the ROIs (head, tail) every 0.033 s in a 1-min period was acquired from fluorescence images of individual *C. elegans* before and after CNS-targeted irradiation. A series of data with 1799 values for luminance of the ROI in head (/tail) was acquired for each dose of irradiation, that is, 0, 500, 1000, or 1500 Gy, before and after irradiation. Data were analyzed by using a linear mixed model (LMM) [22,23,24]. This statistical analysis was performed using MATLAB software. First, data sets regarding Ca^2+^ luminance before and after targeted irradiation were grouped by individuals. In the LMM, Ca^2+^ luminance was fitted by the dose of five levels (after irradiation; 0, 500, 1000, and 1500 Gy; before irradiation (0 Gy) of four doses) and individual-dependent random slope and random intercept were included.

For Ca^2+^ wave analyses, 31 images were extracted every 0.1 s for 3 s from each movie and sorted in order. By focusing on areas with high Ca^2+^ fluorescence in these images, Ca^2+^ wave propagation from head to tail for forward motion was observed.

## 3. Results

### 3.1. CNS-Targeted Irradiation Elicits Dose-Dependent and Irradiated Area-Dependent Effects on Motility

Targeted microbeam irradiation was delivered using a collimating microbeam irradiation system. To target and irradiate the whole nerve ring (representing the entire CNS), we fabricated a new micro-aperture with a 60-µm diameter, corresponding to the body width of *C. elegans*. By using a micro-aperture with 60- and 20-µm diameters, as used in previous CNS-targeted experiments, we addressed irradiated region (tissue)-dependent effects. The CNS-targeted irradiation of a limited 60-µm-diameter area included the entire CNS and body-wall muscle cells around the CNS. Although some pharyngeal muscle cells were also irradiated, pharyngeal muscle cells are not involved in locomotion [11]. In CNS-targeted irradiation limited to a 20-µm-diameter area, the center region of the CNS and the isthmus in the pharynx were irradiated (Figure 1a,c and Table 1). The nerve ring corresponding to the CNS is located in the center of the head and is completely surrounded by body-wall muscle cells. Our microbeam irradiation device irradiates with a vertical beam and, therefore, it is impossible, in principle, to irradiate the CNS without removing the body-wall muscle cells covering the nerve ring. For comparison, we also irradiated the whole body with carbon ions. These experiments enabled us to investigate the tissue-dependent effects of irradiation, that is, effects on the CNS and head muscle cells. Although mammals, including humans, are generally more sensitive to radiation because of their complex body structure and functions, some microorganisms and small animals are extremely resistant to radiation. *C. elegans* is extremely resistant to radiation [4,5,25,26,27,28,29,30,31]. The 80% effective dose (ED_80_) for motility, which represents the dose that reduces motility to 80% of the nonirradiated level, is 272 Gy for whole-body irradiation with high LET radiation, such as heavy ions, and 376 Gy for whole-body irradiation with low LET radiation, such as gamma rays and X-rays [5]. Therefore, we used a dose of heavy-ion irradiation greater than 500 Gy.

In addition, to inhibit free motion during irradiation and to maintain animals in good active condition for long periods, we used a PDMS ion-penetrable, ultra-thin, microfluidic chip (Worm Sheet IR; Figure 1a,b). In previous targeted-irradiation experiments [10], carbon ions could not pass through the conventional microfluidic chip enclosing animals and, therefore, did not reach the ion detector located under the sample stage. This was because the thickness of the microfluidic chip was thicker than the range of carbon ions (approximately 1 mm). In contrast, because the sample thickness (including Worm Sheet-enclosed animals and upper and bottom cover films) was only 550 µm, carbon ions whose range in water is 930 µm could pass through, and the number of ions (dose) applied was carefully controlled [16]. By employing a Worm Sheet, we conducted targeted CNS irradiation in *C. elegans* with an exact number of carbon ion particles (Figure 1). This is the first time that dose responses have been examined by controlling the number of irradiated ions (corresponding to dose of irradiation). The experimental method, including irradiation and locomotion assay, was performed as previously described [10], as shown in Figure 2.

**Figure 5:**
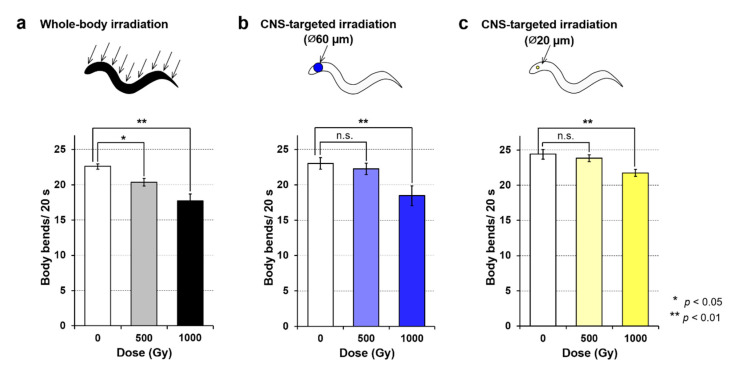
Motility of *C. elegans* immediately after carbon-ion irradiation with a dose of 500 or 1000 Gy. Motility was evaluated using “body bends” [21], defined as the mean value of bends in the anterior body region at 20-s intervals in six animals. (**a**) Motility in animals after whole-body irradiation. Mean values from five independent experiments for whole-body irradiation were calculated for each dose. (**b**) Motility of animals after irradiation of a ∅60-µm micro-aperture region of the CNS. Mean values from five independent experiments using the targeted irradiation of a ∅60-µm micro-aperture region were calculated for each dose. (**c**) Motility of animals after irradiation of a ∅20-µm micro-aperture region of the CNS. Mean values from five independent experiments using targeted irradiation of a ∅20-µm micro-aperture region were calculated for each dose. Error bars represent the SEM of independent experiments. All data were analyzed using one-way ANOVA at 0.05 (*) and 0.01 (**) significance levels.

As shown in Figure 5a, the motility of whole-body irradiated animals was reduced in a dose-dependent manner. The result of whole-body irradiation to animals enclosed in microfluidic channels provided a good reproduction of previous experiments using whole-body irradiation of animals freely moving around on agar [5], confirming that there was no negative effect of on-chip immobilization. When the CNS was targeted with 500 Gy irradiation to a limited 60-µm-diameter area, no significant effect on motility was observed (Figure 5b). Furthermore, when the CNS was targeted with 500-Gy irradiation to a limited 20-µm-diameter area, no effects of irradiation were observed (Figure 5c), consistent with the results of previous irradiation experiments [10]. From these experiments, irradiation with 500 Gy targeted to the CNS within a 60-µm-diameter area does not cause an irradiation area-dependent effect on motility. In contrast, significant effects between nonirradiated control animals were observed with irradiation of 1000 Gy targeted to 60- and 20-µm-diameter areas (Figure 5b–c). Regarding irradiation region (tissue)-dependent effects, the reduction of motility by 1000-Gy irradiation was greater for whole-body irradiation and CNS-targeted irradiation of a 60-µm-diameter area compared with CNS-targeted irradiation of a 20-µm-diameter area. A two-way functional ANOVA with 0.01 significance level indicated that the main effect was observed in the irradiated area (beam size) as well as dose of irradiation, whereas a significant interaction was not found between irradiation area and dose of irradiation. 

These results indicated a dose-dependent effect of CNS-targeted irradiation and an irradiated area-dependent effect of 1000-Gy irradiation on motility. Although a dose of 500 Gy was sufficient to reduce motility using whole-body irradiation, 1000-Gy CNS-targeted irradiation was required to induce the same effects as whole-body irradiation. However, it was unclear whether the effects depended on the beam size or the tissue irradiated. Thus, in subsequent experiments, we focused on the effects on the body-wall muscle cells surrounding the CNS.

### 3.2. Effects of Irradiation on Head Muscle Activities of CNS-Irradiated Animals

To observe only muscle activity in animals in which CNS and body-wall muscle cells around the CNS were irradiated using a 60-µm-diameter micro-aperture, we used the HBR4 strain of *C. elegans* [17], which expresses a reporter gene for the calcium indicator GCaMP3.35 in all body-wall muscle cells used for locomotion. In the HBR4 strain, the contraction strength of body-wall muscle cells during crawling on an agar plate can be determined by the fluorescence intensity of a calcium-ion (Ca^2+^) indicator (Figure 3a). Each body-wall muscle cell emits light periodically from head to tail during forward motion and from tail to head during backward motion; therefore, the direction and speed of locomotion can be easily distinguished by observing the Ca^2+^ waves. For the imaging of body-wall muscle cells, we previously established an on-chip observation method using this strain [18]. The small degree of clearance in the microfluidic channels permits the animals to bend slightly, allowing the observation of muscle contraction and extension during crawling using a calcium-ion indicator (Figure 3b).

**Figure 6:**
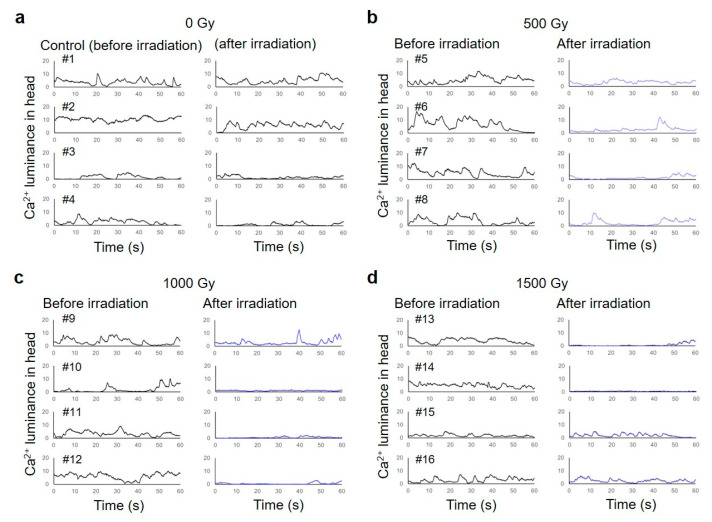
Calcium ion luminance of body-wall muscle cells around the CNS in *C. elegans* HBR4 individuals before and after CNS-targeted irradiation of a ∅60-µm micro-aperture region. Luminance in the ROI (head) of individuals every 0.033 s for 1 min was acquired from fluorescent images before and after irradiation. (**a**) Results of four nonirradiated control animals, #1–#4. “Before irradiation” corresponds to the time immediately after the enclosure of *C. elegans* individuals in microfluidic channels of a microfluidic chip and “after irradiation” corresponds to approximately 3 hours after enclosure. (**b**) Results of four animals, #5–#8, irradiated with a dose of 500 Gy. (**c**) Results of four animals, #9–#12, irradiated with a dose of 1000 Gy. (**d**) Results of four animals, #13–#16, irradiated with a dose of 1500 Gy. The left panels indicate results before irradiation and the right panels indicate results after irradiation.

We applied this novel method for a series of observations of muscle activities of CNS-irradiated animals, as shown in Figure 4. Imaging observations of all animals before irradiation, during CNS-targeted irradiation, and after irradiation were completed on a Worm Sheet microfluidic chip. We compared muscle contraction patterns of the whole body before and after irradiation (Figure 6a–d). We investigated the effect of CNS-targeted irradiation with a dose of 1500 Gy in addition to the doses used in the experiments described in Section 3.1.

As shown in Figure 6a, in four nonirradiated control animals, the luminance of calcium ions (Ca^2+^) corresponding to body-wall muscle contractions in the head for 1 min after irradiation increased and decreased periodically. In most animals, in which a 60-µm-diameter area of the CNS was irradiated with 500, 1000, or 1500 Gy, the frequency of the periodic contraction and relaxation were reduced, and the luminance of Ca^2+^ in the body-wall muscle cells around the CNS was decreased after irradiation (Figure 6b–d). Statistical analysis was performed using a linear mixed model (LMM) [22,23,24]. Table 2 shows that significant negative slopes, β, were observed in activities of body-wall muscle cells around the CNS irradiated with 1000 and 1500 Gy (β_1000Gy_ and β_1500Gy_). These results indicate that irradiation reduced the luminance of Ca^2+^, that is, the activity of the irradiated body-wall muscle cells around the CNS. Furthermore, no significant change was observed in the activities of body-wall muscle cells around the tail after CNS-targeted irradiation (Table 3).

β*_d_* (*d* ∈ {0, 500, 1000, 1500 Gy}) denotes the slope estimated for dose of 0, 500, 1000, and 1500 Gy, respectively.

**Figure 7:**
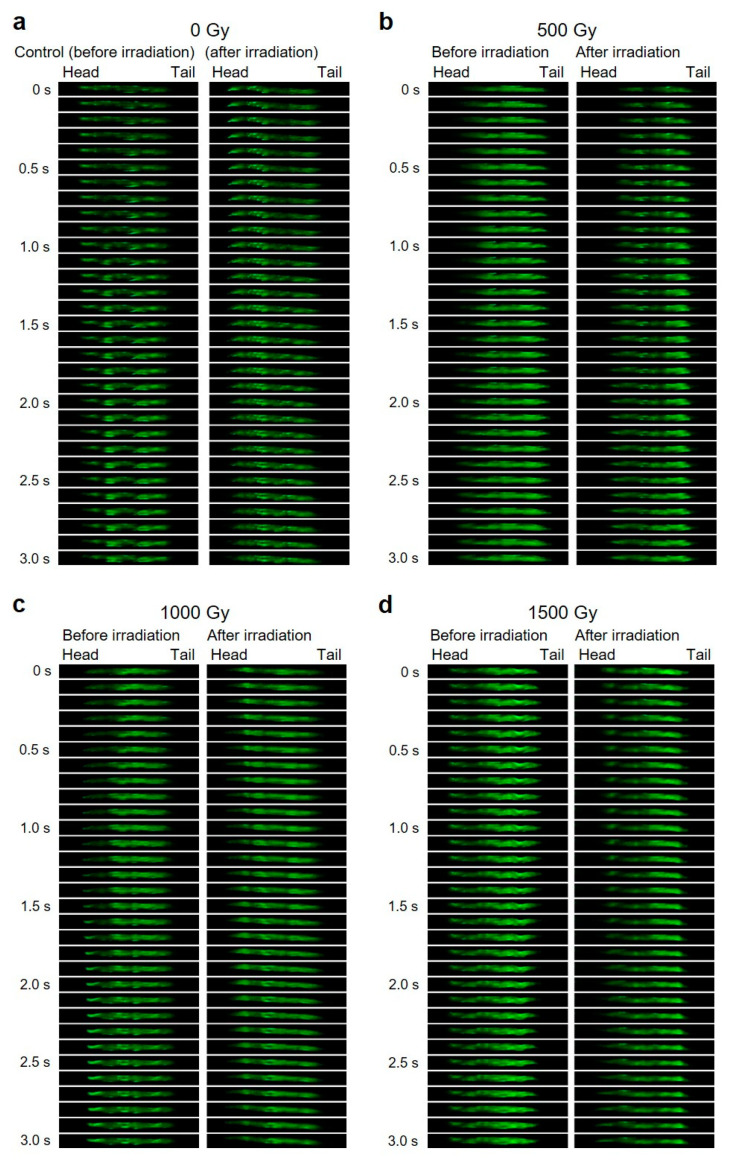
Muscle contraction pattern of body-wall muscle cells in *C. elegans* individuals before and after the CNS-targeted irradiation of a ∅60-µm micro-aperture region. Examples of calcium ion fluorescent images acquired every 0.1 s for 3.0 s from an individual *C. elegans* before and immediately after CNS-targeted irradiation. (**a**) A nonirradiated control animal, which corresponds to #1 in Figure 6a. (**b**) An animal, which corresponds to #5 in Figure 6b, irradiated with a dose of 500 Gy. (**c**) An animal, which corresponds to #9 in Figure 6c, irradiated with a dose of 1000 Gy. (**d**) An animal, which corresponds to #13 in Figure 6d, irradiated with a dose of 1500 Gy. In each image, the head (including the CNS) is on the left.

Additionally, we evaluated the radiation effect on rhythm-pattern generation and propagation of locomotion by observing whole-body muscle activities before and after CNS-targeted irradiation. As shown in Figure 7a, in the nonirradiated control animals, the Ca^2+^ wave was propagated head to tail in a forward motion, corresponding to motor control signals for sinusoidal locomotion (Figure 6a). Similarly, after targeted irradiation with 500, 1000, or 1500 Gy to a limited 60-µm-diameter area, the Ca^2+^ wave occurred from head to tail (Figure 7b–d). These findings were similar to observations before irradiation, although in these situations, Ca^2+^ luminance tended to be decreased slightly in the head region, including the irradiated CNS region. Thus, the rhythm pattern of locomotion can be produced even after irradiation of the CNS and the body-wall muscle cells around the CNS.

From these results, we confirmed that the effects of CNS-targeted irradiation on motility shown in Figure 5 might be induced in part by radiation effects on irradiated body-wall muscle cells around the CNS. The possibility of CNS involvement in the effects of radiation on *C. elegans’* motility remains. On the basis of previous results of targeted CNS irradiation with 500 Gy [10], we concluded that CNS-targeted irradiation does not affect the motility of *C. elegans*; that is, radiation inhibits locomotion by a whole-body mechanism. However, this idea might be revised on the basis of the present experiments, in which the CNS was irradiated with a larger dose of heavy ions or/and to a wider region of the CNS compared with the previous study [10]. Other than irradiation, previous experimental or theoretical studies indicated the involvement of neurons located in the head region, that is, the nerve ring and/or the head part of the nerve cord, in locomotion [12,13,14,32,33,34,35,36], and our findings are consistent with these results.

## 4. Discussion

### 4.1. Effectiveness of PDMS Microfluidic Chips in Targeted-Irradiation Experiments

We established an on-chip immobilization method for microbeam irradiation of live *C. elegans* individuals and successfully conducted region-specific irradiation as previously reported [10]. However, it was not possible to irradiate a sufficiently large number of individuals simultaneously in previous experiments because of the limited capacity of conventional PDMS microfluidic chips. In addition, on-chip immobilization using a conventional microfluidic chip induced drying because of the hydrophobicity of PDMS itself.

To allow irradiation experiments to be conducted more efficiently, we next developed a microfluidic chip with water retention, the Worm Sheet. This microfluidic chip is specialized for simultaneous microbeam irradiation of multiple animals enclosed in a microfluidic chip while maintaining animals in good active condition for a long time [16,18]. In the present study, multiple animals for a single irradiation condition could be simultaneously enclosed in a Worm Sheet and irradiated sequentially. As a result, the efficiency of the experiment was significantly improved compared with previous experiments in which animals were irradiated individually in a microfluidic chip.

Furthermore, we employed on-chip imaging observations, as previously proposed [18]. Animals were enclosed before irradiation and muscle activity based on fluorescence imaging was video recorded. Subsequently, the CNS was irradiated and muscle activity was video recorded immediately after irradiation. Using this method, a series of observations and irradiation was completed in one microfluidic chip. This on-chip observation method enabled us to observe the internal responses of body-wall muscle cells both before and after irradiation, in addition to behavior.

This method is advantageous because each individual could be clearly distinguished and easily observed over time. The behavioral assays in Section 3.1. were based on previous studies and were conducted by collecting animals from microfluidic chips immediately after irradiation. Thus, animals under only a single condition were typically enclosed in each microfluidic chip to avoid the mixing of animals during collection from the microfluidic chip (Figure 2). In contrast, in the on-chip observations in Section 3.2., animals from various irradiation conditions could be enclosed in multiple straight channels on a single microfluidic chip (Figure 4), which dramatically improved experimental efficiency. As shown in Figure 6a and Figure 7a, no adverse effect of on-chip immobilization on muscle activities in nonirradiated control animals was observed, indicating that this method keeps animal in good active condition.

The irradiation experiment presented here was performed in an irradiation facility equipped with a cyclotron. Because of the facility restrictions, a high-performance fluorescence microscope or imaging device could not be brought in for these experiments. Therefore, we used the experimental analysis proposed in a previous study [18], using an observation system that can be implemented with a relatively simple imaging system. In the future, it may be possible to acquire videos with higher resolution.

### 4.2. Effects of Head (CNS)-Targeted Irradiation on Motility Reflect Effects on the CNS and Muscle Cells

As shown in Figure 1a and Table 1, although irradiation of a 20-µm-diameter area only targeted the CNS, the body-wall muscle cells in the head were partially irradiated in addition to the CNS when the targeted irradiation area was 60 µm in diameter. The decrease of Ca^2+^ level in the head immediately after irradiation, identified by comparing Ca^2+^ wave patterns before and after irradiation, corresponded, in part, to the effects on motility. That is, after CNS-targeted irradiation to a limited 60-µm-diameter area, dose-dependent radiation effects on motility were observed (Figure 5b–c). The results indicate that dose-dependent differences in the reduction of motility after CNS-targeted irradiation to a limited 60-µm-diameter area reflect the dose-dependent effects on the CNS and irradiated body-wall muscle cells around the CNS.

The neural mechanism underlying the reduction of locomotion immediately after CNS-targeted irradiation remains unclear, and the possibility that dose-dependent irradiation has a direct effect on the CNS resulting in reduced motility should be investigated in detail in a future study. Especially, conducting molecular- and/or cellular-level analysis of CNS neuron behaviors is the next step. Although we found that cell cycle arrest and apoptosis were induced in nematode germline cells after heavy-ion microbeam irradiation [7], there was no evidence regarding the relationship between the effects of radiation observed at the behavioral level such as locomotion and radiation-induced DNA damage. In addition, it was confirmed that the effects of whole-body irradiation with heavy ions on learning or chemotaxis are smaller than those on motility [5,25,31]. There is a large difference in the radiation resistance between embryos that undergo cell division and adults that have completed cell division [5], indicating DNA damage is not always significant for adult animals that have already finished cell division. However, as we previously reported [4], hydrogen peroxide (H_2_O_2_), a reactive oxygen species (ROS) produced by low and high LET radiation exposure, decreased motility. If the cells at irradiated CNS sites are not undergoing apoptosis (or another type of cell death), then the tentative reduction of motility might be induced by a limited disturbance of signal transduction in the CNS due to radiation-produced ROS.

### 4.3. Remaining Issues on the Targeted Irradiation and Analysis of Cell Responses

A detailed clarification of how CNS function is affected after irradiation is needed in a future study. To better understand CNS involvement in the effects of radiation on the motility of *C. elegans*, we need a more precise microbeam device that can target specific tissue or cells. We could not clearly distinguish whether the reduction of motility after irradiation of a 60-µm-diameter area was caused by effects on specific CNS cells because of the present technical limitations; however, we are developing a novel microbeam device [37] that can irradiate specific neurons and/or muscle cells in the near future.

In addition, animals were not always on their lateral side in the chip during irradiation because the current PDMS microfluidic chip, the Worm Sheet, does not control the orientation of the individual *C. elegans* during irradiation. Thus, animals were irradiated on their dorsal or ventral or left or right sides. Further improvements in microfluidic chips are required to better control targeted irradiation to specific tissues.

Some methods for examining the functions of individual cells and tissues, including mutants and cell function inhibitors, selectively suppress only the functions of arbitrary cells or cell groups. Laser ablation [38,39] and optogenetic ablation [40,41,42,43] can be used to inhibit functions of specific cells. In addition to these established powerful techniques to clarify a tissue responsible for a biological function, we aim to develop a microbeam-based, next-generation method to control responses at cellular or tissue levels. We intend to perturb biological functions using external stimuli, targeted irradiation, and record the responses that differ from normal.

This is the first report of our method and its accuracy is not yet comparable to conventional methods. However, there are inherent advantages in the proposed method and, in the future, it will be an effective tool for analyzing cell and tissue functions.

## 5. Conclusions

This is the first description of CNS-targeted irradiation experiments on animals using a Worm Sheet. The results of the present study provide the first evidence for the involvement of the CNS in radiation-induced reduced motility in adult hermaphrodite *C. elegans* in the context of irradiated area and dose dependency. The findings that motility decreased in a dose-dependent manner immediately after CNS-targeted irradiation, and that the body-wall muscle cells around the CNS may be partly involved, suggest the motor control of *C. elegans* is not independent of the CNS. Our findings are partly consistent with previous experimental or theoretical studies [12,13,14,32,33,34,35,36], which suggested the involvement of neurons located in the head region in locomotion.

## Figures and Tables

**Table 1:** Muscle cells and neurons targeted in whole-body or CNS-targeted irradiation. This was compiled with reference to the information in [1,2,3].

Tissue		Targeted Region		
		Whole Body	CNS (∅60 µm)	CNS (∅20 µm)
Pharyngeal muscle cells	Procorpus	entirely	-	-
	Metacorpus	entirely	partially	-
	Isthmus	entirely	entirely	partially
	Posterior bulb	entirely	partially	-
Body-wall muscle cells in head	Dorsal left medial cells, #3 and #5	entirely	entirely	partially
	Dorsal left lateral, #4 and #6	entirely	entirely	partially
	Ventral left lateral, #4 and #6	entirely	entirely	partially
	Ventral left medial cells, #3 and #5	entirely	entirely	partially
	Ventral right medial cells, #3 and #5	entirely	entirely	partially
	Ventral right lateral cells, #4 and #6	entirely	entirely	partially
	Dorsal right lateral cells, #4 and #6	entirely	entirely	partially
	Dorsal right medial cells, #3 and #5	entirely	entirely	partially
Neurons in the nerve ring	Neurites in the anterior ganglia	entirely	entirely	entirely
	Cell bodies in the anterior ganglia	entirely	entirely	partially
	Neurites in the lateral ganglia	entirely	entirely	entirely
	Cell bodies in the lateral ganglia	entirely	entirely	partially

**Table 2:** Parameters calculated by linear mixed model (LMM) analysis to evaluate changes in activities of the irradiated body-wall muscle cells around the CNS between before and after targeted irradiation.

Coefficients of Fixed Effects				
Coefficient	Estimate	*p*-Value	Lower Limit	Upper Limit
β_0_ (Intercept)	2.34	2.02 × 10^−11^	1.90	2.78
β_0Gy_	0.676	0.277	−0.576	1.93
β_500Gy_	0.0644	0.847	−0.616	0.745
β_1000Gy_	−1.41	0.00761	−2.40	−0.406
β_1500Gy_	−1.19	0.0207	−2.17	−0.195

β*_d_* (*d* ∈ {0, 500, 1000, 1500 Gy}) denotes the slope estimated for dose of 0, 500, 1000, and 1500 Gy, respectively.

**Table 3:** Parameters calculated by LMM analysis to evaluate changes in activities of the body-wall muscle cells around the tail between before and after targeted irradiation.

Coefficients of Fixed Effects				
Coefficient	Estimate	*p*-Value	Lower Limit	Upper Limit
β_0_ (Intercept)	5.23	2.08 × 10^−8^	3.854	6.60
β_0Gy_	−1.14	0.176	−2.817	0.541
β_500Gy_	0.338	0.815	−2.612	3.28
β_1000Gy_	−2.61	0.00137	−4.11	−1.11
β_1500Gy_	−0.877	0.559	−2.16	−3.92

β*_d_* (*d* ∈ {0, 500, 1000, 1500 Gy}) denotes the slope estimated for dose of 0, 500, 1000, and 1500 Gy, respectively.
